# Intermittent Fasting Attenuates Metabolic-Dysfunction-Associated Steatohepatitis by Enhancing the Hepatic Autophagy–Lysosome Pathway

**DOI:** 10.3390/nu15214574

**Published:** 2023-10-27

**Authors:** Kyung Eun Kim, Hyun Joo Shin, Yeajin Ju, Youngae Jung, Hyeong Seok An, So Jeong Lee, Eun Ae Jeong, Jaewoong Lee, Geum-Sook Hwang, Gu Seob Roh

**Affiliations:** 1 Department of Anatomy and Convergence Medical Science, College of Medicine, Institute of Medical Science, Gyeongsang National University, Jinju 52727, Republic of Korea; kke-jws@hanmail.net (K.E.K.); k4900@hanmail.net (H.J.S.); gudtjr5287@hanmail.net (H.S.A.); thwjd5411@naver.com (S.J.L.); jeasky44@naver.com (E.A.J.); woongs1111@gmail.com (J.L.); 2 Integrated Metabolomics Research Group, Western Seoul Center, Korea Basic Science Institute, Seoul 03759, Republic of Korea; juyj0918@naver.com (Y.J.); jya0819@kbsi.re.kr (Y.J.); 3 College of Pharmacy, Chung-Ang University, Seoul 06974, Republic of Korea

**Keywords:** intermittent fasting, autophagy, lysosome, non-alcoholic steatohepatitis

## Abstract

An intermittent fasting (IF) regimen has been shown to protect against metabolic dysfunction-associated steatohepatitis (MASH). However, the precise mechanism remains unclear. Here, we explored how IF reduced hepatic lipid accumulation, inflammation, and fibrosis in mice with MASH. The mice were fed a high-fat diet (HFD) for 30 weeks and either continued on the HFD or were subjected to IF for the final 22 weeks. IF reduced body weight, insulin resistance, and hepatic lipid accumulation in HFD-fed mice. Lipidome analysis revealed that IF modified HFD-induced hepatic lipid composition. In particular, HFD-induced impaired autophagic flux was reversed by IF. The decreased hepatic lysosome-associated membrane protein 1 level in HFD-fed mice was upregulated in HFD+IF-fed mice. However, increased hepatic lysosomal acid lipase protein levels in HFD-fed mice were reduced by IF. IF attenuated HFD-induced hepatic inflammation and galectin-3-positive Kupffer cells. In addition to the increases in hepatic hydroxyproline and lumican levels, lipocalin-2-mediated signaling was reversed in HFD-fed mice by IF. Taken together, our findings indicate that the enhancement of the autophagy–lysosomal pathway may be a critical mechanism of MASH reduction by IF.

## 1. Introduction

High-fat diet (HFD)-induced weight gain causes insulin resistance and promotes the spectrum of liver diseases known as nonalcoholic fatty liver disease (NAFLD), recently renamed metabolic-dysfunction-associated steatotic liver disease (MASLD) [1]. An increased triglyceride (TG) level in hepatocytes is the primary feature of MASLD [2]. MASLD can comprise simple steatosis or more advanced metabolic-dysfunction-associated steatohepatitis (MASH), which is characterized by inflammation and hepatic ballooning injury accompanied by fibrosis [3,4]. In particular, disrupted hepatic lipid homeostasis, resulting in TG accumulation in hepatocytes, is a hallmark of MASLD [5]. This excessive lipid accumulation exceeds the liver’s capacity to store, secrete, and oxidize fatty acids [6]. Hepatic inflammation and fibrosis induced by lipotoxicity promote MASH. Therefore, regulation of the hepatic TG level is essential to prevent or reverse the progression of MASH. However, there are no specific therapeutic strategies to counteract lipotoxicity in MASH, because its pathogenesis is complicated and is still poorly understood.

The liver stores neutral lipids such as TGs in specialized single-membrane vacuoles known as lipid droplets (LDs) [7]. Increased lipolytic activity in the adipose tissue is associated with the primary fatty acid source that is taken up by the liver [8]. Twenty-six percent of the TGs stores in the liver result from increased de novo lipogenesis, and only 15% result from dietary intake. Furthermore, increased hepatic lipid accumulation can also be caused by impaired lipophagy [9]. In a lipidomic analysis, differences were found in the abundance of specific lipid species between healthy subjects and MASLD patients [10,11]. In particular, low levels of phosphatidylcholines (PCs) and phosphatidylethanolamines (PEs) may contribute to the progression of MASH by initiating inflammation or increasing the formation of large LDs [12]. However, little is known about whether altered lipid profiles within hepatocytes influence inflammation and fibrosis in MASH.

In addition to drug therapy for MASLD, physical activity and dietary restriction reduce body weight and enhance insulin sensitivity in patients with MASLD. Our previous studies demonstrated that caloric restriction attenuated MASLD in HFD-fed, ob/ob, and db/db mice [13,14,15]. Caloric restriction, by providing approximately 60–70% of the normally consumed food, significantly reduces body weight and insulin resistance. However, intermittent (alternate-day) fasting (IF) is a general lifestyle intervention that contributes to the prevention of MASH progression [16]. Interestingly, IF for 20 weeks had neuroprotective effects against excitotoxic stimuli [17]. However, the roles of long-term IF in hepatic lipid accumulation, lipophagy, inflammation, and fibrosis in MASH are unknown.

Based on this background, in this study, we employed lipidomic analysis of altered hepatic lipid profiles and transmission electron microscopy in an HFD-induced MASH mouse model to clarify the mechanisms underlying impaired lipophagy. In particular, we demonstrated the protective effects of long-term IF against impaired lipophagy, inflammation, and fibrosis in HFD-fed mice. These findings suggest that the autophagy–lysosome pathway in hepatic LDs may play an important role in the development of MASLD and its progression to MASH, which is correlated with inflammation and fibrosis.

## 2. Materials and Methods

### 2.1. Animals and IF Mouse Model

Three-week-old male C57BL/6 mice were purchased from KOATECH (Pyeongtaek, Republic of Korea). The mice (*n* = 12 per group) were divided into normal diet (ND), HFD (60% kcal from fat, Research Diets Inc., New Brunswick, NJ, USA), HFD + IF (HIF), and ND + IF (NIF) groups. Mice in the ND and HFD groups were fed an ND or an HFD for 30 weeks, whereas mice in the HIF group were fed an HFD for 8 weeks and then switched to an IF protocol consisting of alternating 24 h periods of fasting and feeding for 22 weeks. All mice were fasted overnight before sacrifice at 34 weeks of age. The mice were individually housed under an alternating 12 h light/dark cycle. The mice were weighed monthly, and the fasting blood glucose level was measured from 12 weeks of age using an Accu-Chek glucometer (Roche Diagnostics GmbH, Mannheim, Germany).

### 2.2. Measurement of Serum Metabolic Parameters

Using Zoletil (20 mg/kg, Virbac Laboratories, Carros, France) and Rompun (5 mg/kg, Bayer, Bayer Korea, Republic of Korea), the mice were anesthetized following an overnight fasting. After being removed from the left ventricle, blood samples were centrifuged. The levels of serum alanine aminotransferase (ALT), aspartate aminotransferase (AST), and total cholesterol were measured at the Green Cross Reference Laboratory in Yougin, Republic of Korea. A mouse insulin enzyme-linked immunosorbent assay kit (Shibayagi Co., Gunma, Japan) was used to measure serum insulin.

### 2.3. Tissue Collection and Histological Analysis

The mice (*n* = 4 per group) were perfused with 0.1 M phosphate-buffered saline (PBS) containing 4% paraformaldehyde (PFA). The pancreas and liver were fixed in 4% PFA for 12 h at 4 °C, embedded in paraffin, and cut into 5-μm sections. Hematoxylin- and eosin (H&E, Abcam, Cambridge, MA, USA)-stained slides were visualized using a BX53 microscope (Olympus, Tokyo, Japan). Frozen sections of liver were stained with Nile Red (Sigma-Aldrich, St. Louis, MO, USA) in the dark for 10 min and counterstained with 4′, 6-diamidino-2-phenylindole (DAPI, Invitrogen, Carlsbad, CA, USA). The percentage of Nile Red-positive area (250 × 250 µm^2^) in three sections was measured using i-Solution version 8.0 software (IMT i-Solution, Inc., Vancouver, BC, Canada). For histological fibrosis, Picro-Sirius Red (Sigma-Aldrich)-positive areas (5–14 fields) were quantified using ImageJ software (version 1.52a, NIH, Bethesda, MD, USA).

### 2.4. MASLD Activity Score Measurement

Using H&E-stained slides, the MASLD activity score was defined as an unweighted sum of scores for liver steatosis (0–3), lobular inflammation (0–3), and hepatocyte ballooning (0–2) [18].

### 2.5. Hepatic TG Colorimetric Assay

Frozen livers (*n* = 6 per group) were used to determine TG levels from using a TG colorimetric assay kit (Cayman Chemical Company, Ann Arbor, MI, USA).

### 2.6. Hepatic Hydroxyproline Assay

We measured the hepatic hydroxyproline concentration (*n* = 4–6 per group) using a mouse hydroxyproline assay kit (Cell Biolabs, Inc., San Diego, CA, USA).

### 2.7. Lipid Profiling Analysis

Liver samples (60 mg, *n* = 6 per group) were extracted with 600 μL of a chloroform–methanol–water (2:1:1, *v*/*v*) solution and dried under nitrogen gas. Lipid extracts were reconstituted in a 200 μL isopropanol solution. Lastly, the UPLC/Q-TOF MS system was filled with a 2 μL injection of the solution. Using the Waters ACQUITY UPLC system (Waters, Manchester, UK) with a triple TOF 5600 Mass Spectrometer (Sciex, Concord, ON, Canada), lipid profiling was carried out. As previously described [19], the positive- and negative-ion modes for separation were performed with a Waters CSH (2.1 × 100 mm) with 1.7-μm particles (Waters). Accurate mass measurements for each peak were corrected after acquisition using LockSpray (leucine-enkephalin solution; *m*/*z* 556.28). Progenesis QI version 3.0 software (Waters) was used to analyze the spectral data in order to identify peaks, perform the alignment, and produce peak tables of *m*/*z* and retention times. Standard compounds and online databases (HMDB, LIPID MAPS) were used to identify lipids.

### 2.8. RNA Isolation and Real-Time Polymerase Chain Reaction (RT-PCR) Analysis

Liver tissues (*n* = 4–5 per group) were homogenized in TRIzol reagent (Invitrogen, Carlsbad, CA, USA) to extract total RNA according to the manufacturer’s protocol. The total RNA was then reverse transcribed into cDNA using the RevertAid First Strand cDNA Synthesis Kit (Thermo Fisher Scientific, Carlsbad, CA, USA). RT-PCR was performed using a LightCycler 480 Instrument II (Roche Diagnostics GmbH, Mannheim, Germany). PCR amplification was performed using iQ™ SYBR Green Supermix (Bio-Rad, Hercules, CA, USA) with specific primers (Supplementary Table S1). Relative quantification was performed using the ∆∆Ct method. The relative mRNA expression was expressed as the fold change relative to a calibrator sample. All samples were run in duplicate, and average values were calculated.

### 2.9. Western Blot Analysis

Frozen livers (*n* = 3–8 per group) were homogenized in T-PER lysis buffer (Thermo Fisher Scientific) with a protease and phosphatase inhibitor cocktail (Thermo Fisher Scientific). A BCA assay (Thermo Fisher Scientific) was used to determine the protein concentration. The primary antibodies used are shown in Supplementary Table S2. β-Actin was used as a loading control to normalize protein levels. Protein bands were detected using an enhanced chemiluminescence substrate (Pierce, Rockford, IL, USA). The band density was analyzed using the Multi-Gauge V 3.0 (Fujifilm, Tokyo, Japan) image analysis application.

### 2.10. Immunohistochemistry

Pancreatic sections were placed in 0.3% H_2_O_2_ for 30 min, washed, and incubated in 5% donkey serum for 1 h and then incubated with primary antibody (Supplementary Table S2) at 4 °C overnight and with secondary biotinylated antibody for 1 h. After three washes, the sections were developed with an avidin–biotin–peroxidase complex solution (Vector Laboratories, Burlingame, CA, USA) with a 0.05% diaminobenzidine substrate kit (Vector Laboratories). The sections were then dehydrated in graded alcohol solutions followed by xylene and mounted under coverslips with Permount (Sigma–Aldrich). Insulin-positive areas (200 × 200 µm^2^) in three sections were measured using i-Solution version 8.0 software (IMT i-Solution, Inc.).

### 2.11. Double Immunofluorescence

Paraffin-embedded liver sections were incubated with primary antibodies (Supplementary Table S2) and corresponded to an Alexa Fluor 488- or 594-conjugated secondary antibody. Nuclei were counterstained with DAPI. Representative images from slides were obtained using a BX51-DSU microscope (Olympus).

### 2.12. Transmission Electron Microscopy (TEM)

The mice (*n* = 2 per group) were anesthetized and perfused with both 2% PFA and 2% glutaraldehyde in 0.1 M PBS. The liver was fixed using the same fixative solution for 18 h at 4 °C. Then, the sections were rinsed with 0.1 M PBS and osmicated in 1% osmium tetroxide for 1.5 h. The sections were then dehydrated in graded alcohols, infiltrated with propylene oxide for 10 min, and embedded using a Poly/Bed 812 kit (Polysciences, FL, USA) for 18 h. Sections were cut at 60 nm with a diamond knife and stained with 5% uranyl acetate for 10 min and 1% lead citrate for 5 min. Images were obtained using a transmission electron microscope (JEM-1011, JEOL Ltd., Tokyo, Japan) at an 80-kV accelerating voltage and were photographed with a digital CCD camera (EMSIS GmbH, Muenster, Germany).

### 2.13. Statistical Analysis

Statistical analyses were performed using PRISM 7.0 (GraphPad Software Inc., San Diego, CA, USA). Group differences were determined using two-way analysis of variance (ANOVA) followed by Tukey’s post hoc test. All values are expressed as the mean ± standard error of the mean (SEM). A *p*-value < 0.05 was considered statistically significant.

## 3. Results

### 3.1. IF Reduces Body Weight and Insulin Resistance in HFD-Fed Mice

We measured the changes in mice given an HFD for 20 weeks to investigate the effects of IF on body weight and blood glucose levels. From 16 weeks of age, after 4 weeks of IF, both body weight and fasting blood glucose were significantly reduced in HFD-fed mice (Supplementary Figure S1a,b). To determine whether IF affects circulating insulin levels, we measured serum insulin levels and insulin-positive areas in pancreatic tissue. The increased serum insulin level in HFD-fed mice was significantly reduced by IF (Supplementary Figure S1c). Consistent with the effects of IF on circulating insulin, IF attenuated the increased insulin-positive pancreatic islet area in HFD-fed mice (Supplementary Figure S1d,e). These results indicate that IF reduces body weight and improves HFD-induced insulin resistance.

### 3.2. IF Attenuates MASLD Activity in HFD-Fed Mice

**Figure 1:**
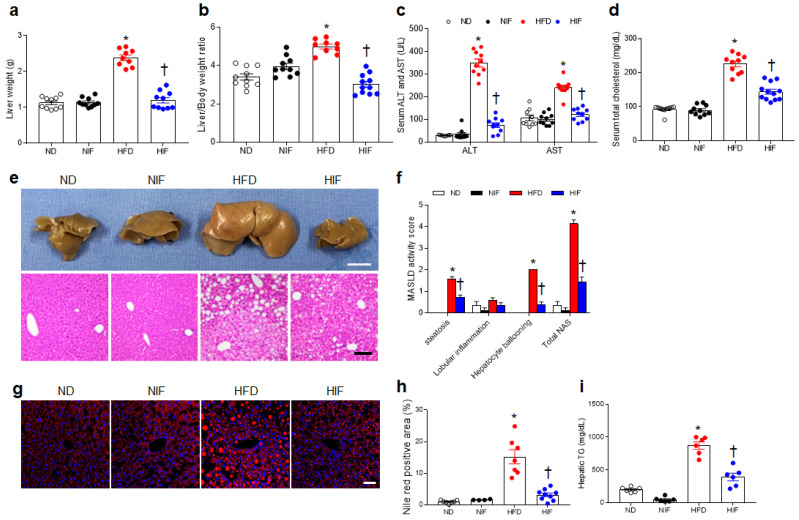
IF attenuates MASLD activity in HFD-fed mice. Male C57BL/6 mice were fed an ND or an HFD for 30 weeks, whereas mice in the NIF or HIF group were fed an ND or HFD for 8 weeks and then were switched to an IF protocol consisting of alternating 24 h periods of fasting and feeding for 22 weeks. (**a**) Liver weight, (**b**) Liver/Body weight ratio, (**c**) serum ALT and AST, and (**d**) serum total cholesterol levels are shown. (**e**) Representative images of livers and H&E staining of liver sections. Scale bars, 1 cm (upper), 100 µm (lower). (**f**) MASLD activity score. (**g**) Representative Nile Red staining with DAPI of liver sections. Scale bar, 50 µm. (**h**) Nile Red-positive area. (**i**) Hepatic TG levels. Significance was determined by two-way ANOVA. * *p* < 0.05 vs. ND. † *p* < 0.05 vs. HFD.

Consistent with the body weight changes over 20 weeks, IF significantly reduced HFD-induced liver weight and the liver/body weight ratio (Figure 1a,b). HIF-fed mice had lower serum hepatic enzymes (ALT and AST) and total cholesterol levels than did HFD-fed mice (Figure 1c,d). Consistent with the change in liver weight, gross liver pathology and histological analysis revealed that HIF-fed mice exhibited reduced MASLD activity scores compared with those in HFD-fed mice (Figure 1e,f). In addition to the hepatic TG levels, histological Nile Red staining revealed decreased lipid accumulation in the liver tissue of HIF-fed mice compared with that in HFD-fed mice (Figure 1g–i). In particular, IF did not affect these metabolic parameters in NIF-fed mice. These results indicate that IF reduces hepatic lipid accumulation and inhibits MASLD.

### 3.3. IF Reduces Hepatic Lipid Uptake and Improves Fatty Acid Oxidation in HFD-Fed Mice

**Figure 2:**
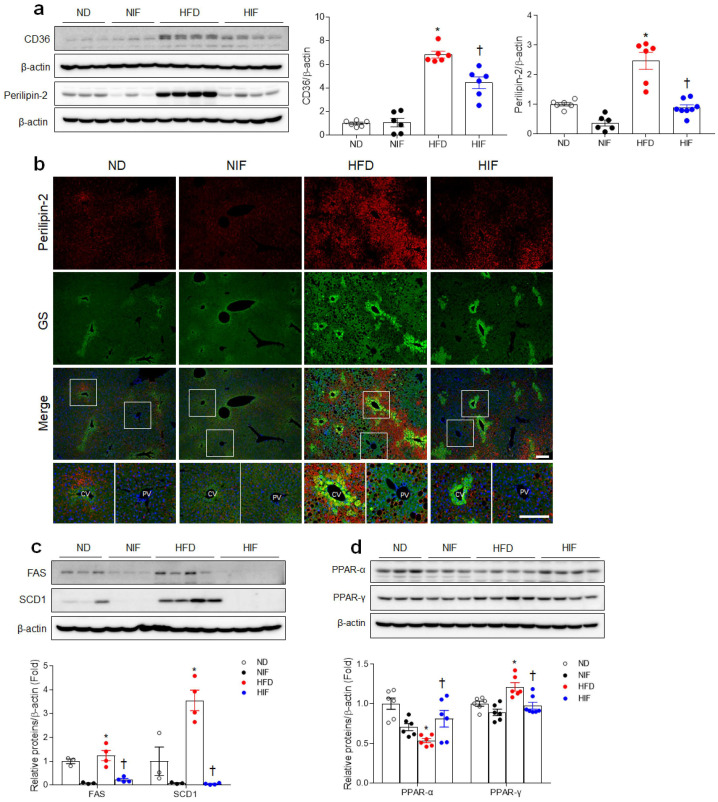
IF reduces hepatic lipid uptake and fatty acid oxidation in HFD-fed mice. (**a**) Western blot analysis and quantification of GS and perilipin-2 protein in liver lysates. (**b**) Representative perilipin-2 and GS staining of liver sections. The highly magnified images in the white boxes within the merged panels are shown in the bottom panels. Scale bars, 50 µm. (**c**,**d**) Western blot analysis and quantification of FAS, SCD1 (**c**), PPAR-α, and PPAR-γ (**d**) protein in liver lysates. β-Actin was used as a loading control. Significance was determined by two-way ANOVA. * *p* < 0.05 vs. ND. † *p* < 0.05 vs. HFD.

CD36/fatty acid translocase is closely associated with the progression of MASLD, which increases with hepatic TG content [20]. Therefore, we hypothesized that alternative HFD feeding would reduce hepatic CD36 levels in HIF-fed mice. As expected, IF prominently attenuated HFD-induced CD36 protein expression (Figure 2a). Additionally, HFD-induced hepatic perilipin-2 protein, a widely expressed cytoplasmic LD scaffolding protein, was reduced by IF (Figure 2a). We performed double immunofluorescence staining to determine which liver zone accumulates hepatic LDs in HFD-fed mice (Figure 2b). Many glutamine synthetase (GS)-specific pericentral hepatocytes were observed in perilipin-2-positive hepatocytes in HFD-fed mice. However, HIF-fed mice showed fewer perilipin-2-stained pericentral hepatocytes. To determine whether IF affects de novo lipogenesis in HFD-fed mice, hepatic fatty acid synthase (FAS) and stearoyl-CoA desaturase 1 (SCD1) protein levels were evaluated using Western blot analysis. IF significantly reduced HFD-induced FAS and SCD1 protein levels (Figure 2c). We found that decreased hepatic peroxisome proliferator-activated receptor (PPAR)-α and increased PPAR-γ levels in HFD-fed mice were reversed by IF (Figure 2d). Taken together, these findings suggest that reducing lipid uptake and lipogenesis and enhancing fatty acid oxidation may be a key mechanism of MASLD reduction by IF.

### 3.4. IF Alters Lipid Profiles in Livers with MASLD

**Figure 3:**
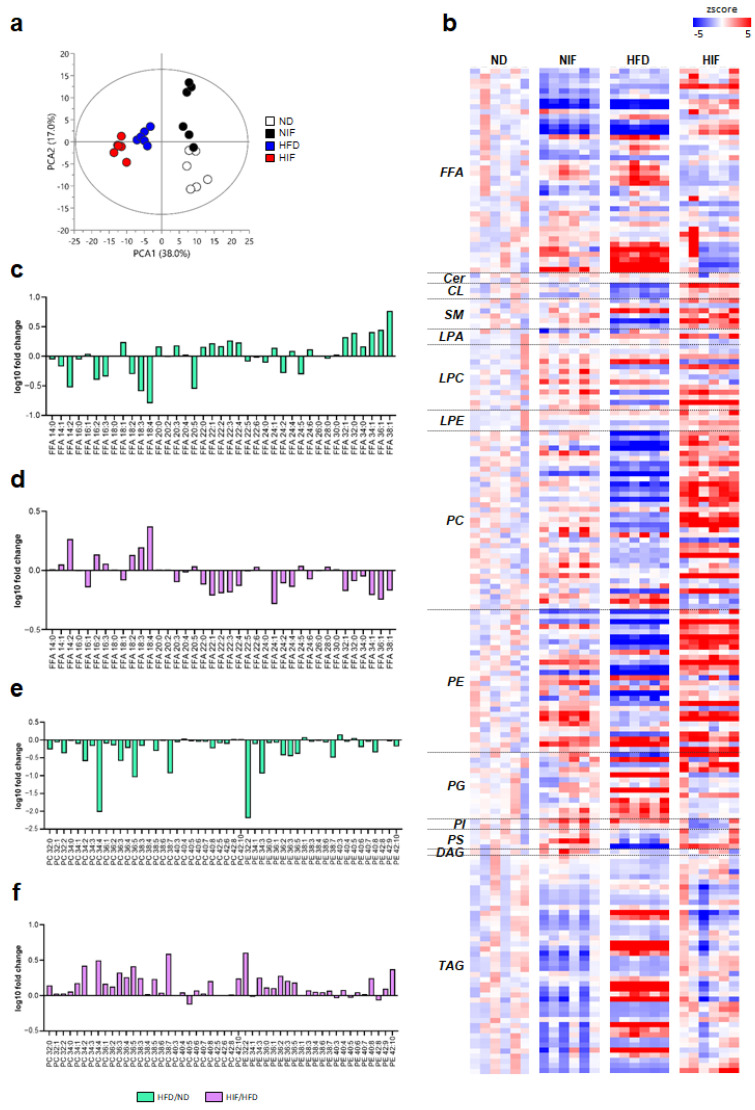
Lipidomic profiling of liver tissues from ND, NIF, HFD, and HIF mice. (**a**) PCA score plot of lipids from liver tissues (R^2^X: 71.2%, Q^2^: 47.3%). (**b**) Heatmaps of lipids identified in liver tissues. The heatmaps are color coded based on the z-scores of the measured relative intensities of each sample. (**c**) Log10-fold changes in FFAs in the liver tissues of ND- and HFD-fed mice. (**d**) Log10-fold changes in FFAs in the liver tissues of HFD- and HIF-fed mice. (**e**) Log10-fold changes in phospholipids in the liver tissues of ND- and HFD-fed mice. (**f**) Log10-fold changes in phospholipids in the liver tissues of HFD- and HIF-fed mice.

Lipidomic analysis using UPLC/Q-TOF MS in both positive- and negative-ion modes was used to determine the hepatic lipidome profiles (Supplementary Figure S2a,b). Triacylglycerol, diacylglyceride, sphingomyelin, ceramide, and cardiolipin were detected in the positive-ion mode, and free fatty acid (FFA), PC, PE, phosphatidylinositol, phosphatidic acid, phosphatidylglycerol, and phosphatidylserine were detected in the negative-ion mode. The precise mass, isotope patterns, MS/MS fragmentation data, and retention time were used to identify the different species of lipids. Multivariate analysis was performed to determine the tendency of hepatic lipidomic alterations in mice that underwent IF. The principal component analysis (PCA) score plot derived from the identified lipids showed a clear separation among the four groups (Figure 3a). A heatmap used to evaluate lipid patterns showed that the lipid levels in HFD-fed mice were opposite to those in HIF-fed mice (Figure 3b and Supplementary Table S3). FFA, PC, and PE levels presented characteristic changes in the hepatic lipidome of HFD-fed and HIF-fed mice. Log10-fold changes between ND-fed and HFD-fed mice and between HFD-fed and HIF-fed mice were used to identify the levels of each FFA chain, PC, and PE (Figure 3c–f). Most FFA species with short chain lengths (<20 carbon atoms) had negative log10-fold changes, corresponding to decreased levels, and most FFA species with long chain lengths (>22) had positive log10-fold changes, corresponding to increased levels in HFD-fed mice compared to those in ND-fed mice (Figure 3c). Conversely, HIF vs. HFD-fed mice had an opposite FFA species pattern compared with that in HFD vs. ND-fed mice (Figure 3d). Furthermore, among hepatic FFAs, IF did not affect saturated fatty acid levels in HFD-fed mice, whereas monounsaturated fatty acid (MUFA) and polyunsaturated fatty acid (PUFA) levels were reversed by IF (Supplementary Figure S2c). The levels of PC and PE species mostly decreased in HFD-fed mice compared to ND-fed mice (Figure 3e), and mostly increased in HIF-fed mice compared to HFD-fed mice (Figure 3f). The increased PC/PE ratio in HFD-fed mice was also reversed by IF (Supplementary Figure S2d). In particular, IF significantly reduced HFD-induced phosphatidylethanolamine N-methyltransferase (PEMT), a transferase enzyme that converts PE into PC (Supplementary Figure S2e). Therefore, this lipidomic analysis indicated that IF could modify the hepatic lipid composition in HFD-induced MASLD.

### 3.5. IF Improves Hepatic Autophagy Flux and Enhances Lysosomes in HFD-Fed Mice

**Figure 4:**
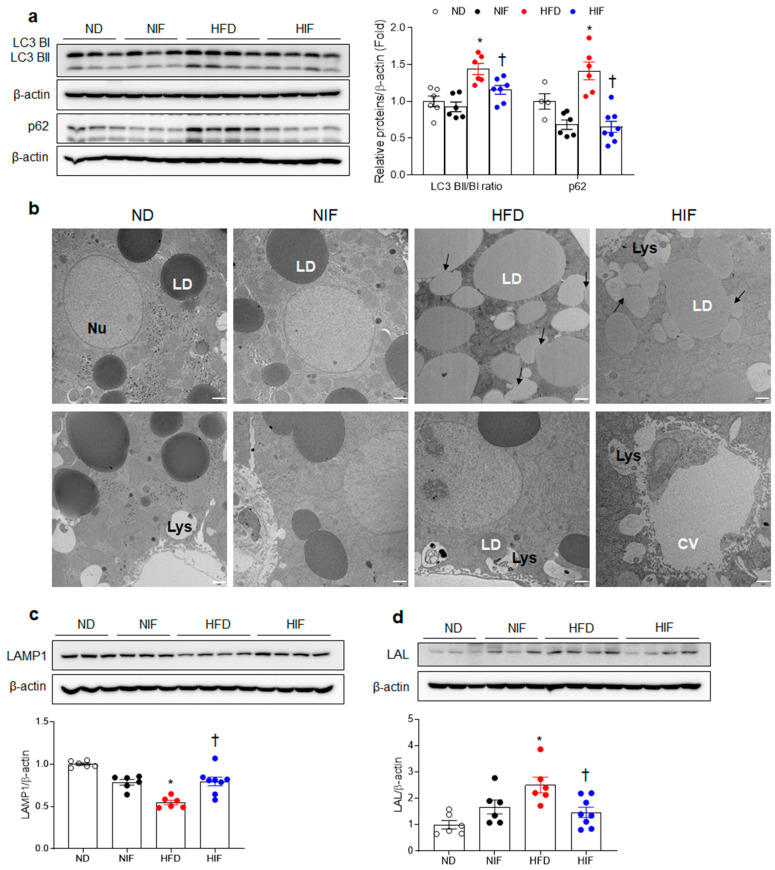
IF improves hepatic autophagy flux and enhances lysosomes in HFD-fed mice. (**a**) Western blot analysis and quantification of LC3B and p62 protein in liver lysates. (**b**) Electron micrographs of hepatocytes. Arrows indicate regions of contact between the lysosomal and LD compartment (Nu, nucleus; LD, lipid droplet; Ly, lysosome; CV, central vein). Scale bar, 1 µm. (**c**,**d**) Western blot analysis and quantification of LAMP1 (**c**) and LAL (**d**) protein levels in liver lysates. β-Actin was used as a loading control. Significance was determined by two-way ANOVA. * *p* < 0.05 vs. ND. † *p* < 0.05 vs. HFD.

Given that LC3 lipidation and its correlation with autophagosome membranes have been demonstrated to be valuable signs of autophagy, we investigated autophagic flux and lysosome enhancement in HFD-fed mice. As expected, significant increases in the LC3BⅡ/Ⅰratio and p62 were detected in the livers of HFD-fed mice compared with those in ND-fed mice (Figure 4a). However, IF reversed these protein levels. These results indicate that a significant increase in p62 in HFD-fed mice indicates impairment of the autophagic flux. Evaluation of the autophagy–lysosome pathway was performed at the ultrastructural level in liver sections processed for TEM (Figure 4b). Increased amounts of LDs, lipolysosomes (LD-loaded lysosomes), and droplet fusion were found in HFD-fed mice compared with those in ND or NIF-fed mice. Fatty acids generated from lysosomal lipolysis can be effluxed from hepatocytes as a result of lysosomal fusion to the plasma membrane and the expulsion of luminal contents. Interestingly, many lipolysosomes fused to the plasma membrane of hepatocytes and effluxed into the central vein (CV) in HIF-fed mice were comparable to those in HFD-fed mice (Figure 4b). In addition, we found that the decreased lysosomal-associated membrane protein 1 (LAMP1) protein level in HFD-fed mice was significantly reversed by IF (Figure 4c). Lysosomal acid lipase (LAL) has been shown to aid in the degradation of LDs through the lysosomal–autophagy pathway [21]. The hepatic LAL protein level was higher in HFD-fed mice than in HIF-fed mice (Figure 4d). Taken together, these findings suggest that an HFD impairs autophagy flux and lysosomes and causes the accumulation of LDs in hepatocytes, whereas IF enhances this impaired autophagy flux–lysosome pathway.

### 3.6. IF Reduces Hepatic Inflammation in HFD-Fed Mice

**Figure 5:**
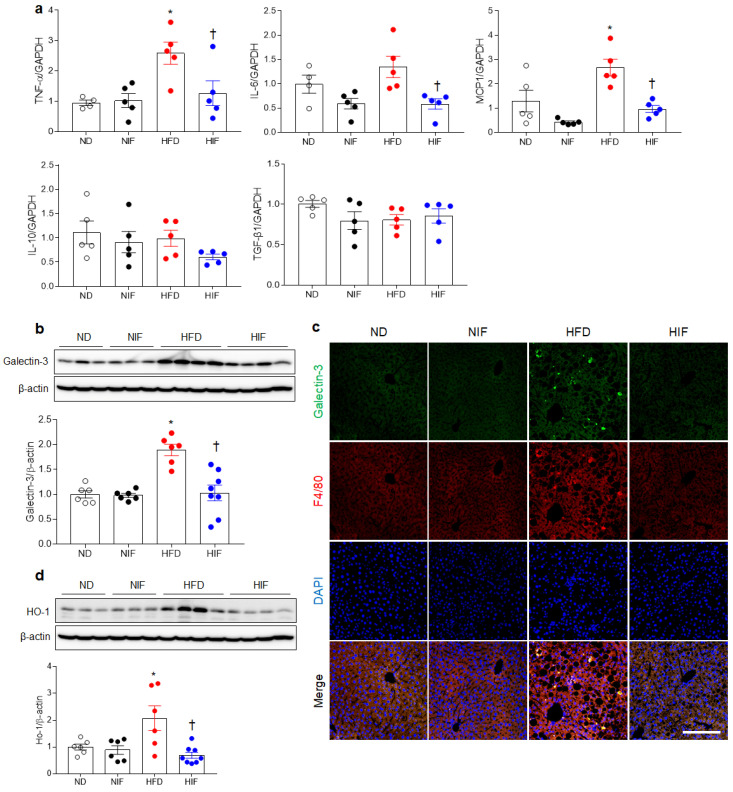
IF reduces hepatic inflammation in HFD-fed mice. (**a**) Proinflammatory cytokines and anti-inflammatory cytokines. (**b**) Western blot analysis and quantification of galectin-3 protein in liver lysates. (**c**) Representative galectin-3 and F4/80 staining of liver sections. Nuclei were counterstained with DAPI. Scale bar, 50 µm. (**d**) Western blot analysis and quantification of HO-1 protein in liver lysates. β-Actin was used as a loading control. Significance was determined by two-way ANOVA. * *p* < 0.05 vs. ND. † *p* < 0.05 vs. HFD.

Hepatic lipid accumulation and impaired lipophagy promote hepatic inflammation [22]. We first measured the mRNA levels of several cytokines including tumor necrosis factor (TNF)-α, interleukin (IL)-6, monocyte chemoattractant protein (MCP)1, IL-10, and transforming growth factor (TGF)-β1 using RT-PCR. IF significantly attenuated HFD-induced changes in proinflammatory cytokines (TNF-α, IL-6, and MCP1), but anti-inflammatory cytokines (IL-10 and TGF-β1) were not altered (Figure 5a). Because galectin-3 is increased in mice with MASH and hepatic fibrosis [23,24], Western blot and immunofluorescence assays were performed (Figure 5b,c). As expected, Western blot analysis showed that IF reduced HFD-induced galectin-3 levels in HFD-fed mice (Figure 5b). Double immunofluorescence revealed that increased colocalization between galectin-3 and F4/80-positive Kupffer cells in HFD-fed mice was attenuated by IF (Figure 5c). Additionally, IF inhibited the HFD-induced increase in the heme oxygenase-1 (HO-1) protein level (Figure 5d). These results indicate that IF can attenuate Kupffer cell-mediated inflammation in mice with MASLD.

### 3.7. IF Reduces Hepatic Fibrosis in HFD-Fed Mice

**Figure 6:**
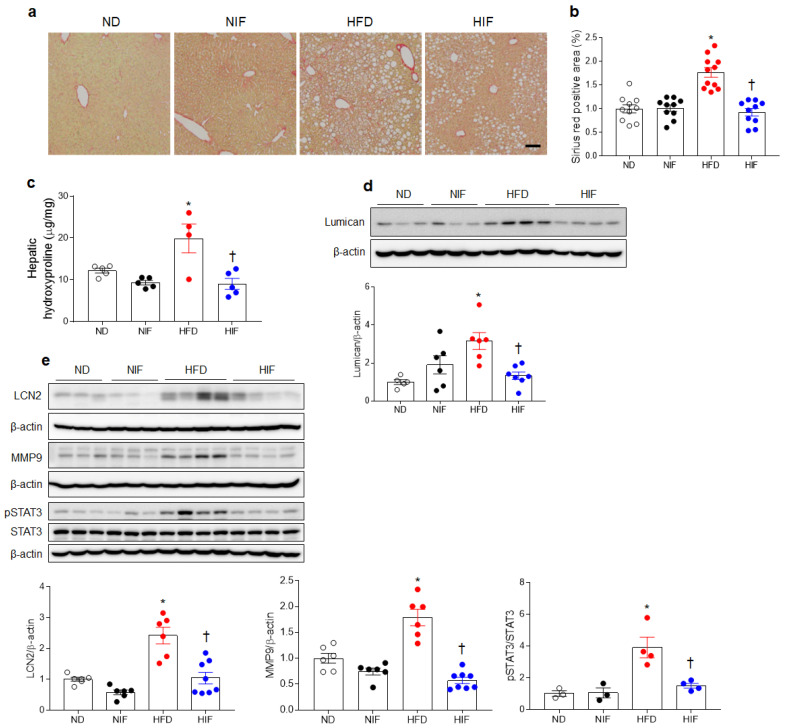
IF reduces hepatic fibrosis in HFD-fed mice. (**a**,**b**) Representative images (**a**) and quantification (**b**) of Picro-Sirius Red staining in liver sections. Scale bar, 100 µm. (**c**) Hepatic hydroxyproline concentration. (**d**,**e**) Western blot analysis and quantification of lumican (**d**), LCN2, MMP9, pSTAT3, and total STAT3 (**e**) proteins in liver lysates. β-Actin was used as a loading control. Significance was determined using a two-way ANOVA. * *p* < 0.05 vs. ND. † *p* < 0.05 vs. HFD.

Hepatic lipid accumulation and inflammation promote progression from MASLD to MASH [25]. Histological analysis showed that IF significantly attenuated Picro-Sirius Red staining, indicating fibrotic changes in HFD-fed mice (Figure 6a,b). Furthermore, HFD-induced hepatic levels of the extracellular matrix proteoglycans hydroxyproline and lumican were markedly reversed by IF (Figure 6c,d). Given that LCN2 is associated with hepatic fibrosis in HFD-fed *ob/ob* mice [26], we examined the effects of IF on the LCN2-related signaling pathway in HFD-fed mice (Figure 6e). Increased hepatic LCN2/matrix metalloproteinase 9 (MMP9)/phosphorylated signal transducer and activator of transcription 3 (pSTAT3) protein expression in HFD-fed mice was markedly reversed by IF. These findings suggest that IF could reduce hepatic fibrosis via LCN2-mediated signaling in mice with MASH.

## 4. Discussion

This study demonstrated that long-term HFD feeding causes insulin resistance and dramatic alterations in hepatic lipid profiles. Impaired autophagic flux promoted the progression to MASH along with inflammation and fibrosis. However, we determined the potential mechanism by which IF attenuates MASH via reduction in hepatic lipid uptake and impairing autophagic flux. Therefore, we suggest that excessive hepatic lipid accumulation and lipogenesis exacerbate hepatocyte damage and that the progression of MASH along with hepatic inflammation and fibrosis can be reduced or reversed by IF.

The IF regimen of alternating 24 h periods of fasting and feeding for 20 weeks improved insulin resistance and hepatic damage. In addition to reductions in liver weight and MASLD activity, IF attenuated hepatic TG and lipid accumulation in HFD-fed mice. Our data indicate that, although alternating HFD feeding for 20 weeks caused a mild increase in serum total cholesterol and hepatic TG levels, MASH progression can be inhibited by this dietary method.

Numerous studies have shown that MASLD is associated with increased hepatic TG content [10,11]. There are generally four major pathways for hepatic TG accumulation, including upregulated FFA uptake from diet or adipose tissue lipolysis, de novo lipogenesis, decreased fatty acid oxidation, and low levels of TG secretion [5,9]. In this study, we confirmed that IF reduces lipid uptake via CD36 and increases fatty acid oxidation by upregulating PPAR-α. In particular, GS-specific pericentral hepatocytes in HFD-fed mice had perilipin-2-positive LDs, but perilipin-2-hepatocytes were not present in HIF-fed mice. This finding supports that LD accumulation in zone 3 of the liver can primarily cause hepatic damage including steatosis, lobular inflammation, and hepatocyte ballooning in HFD-fed mice. However, perilipin-2 is associated with promoting lipid accumulation and inflammation in MASH [27,28]. Najt et al. demonstrated that liver-specific perilipin-2 deletion alleviates methionine–choline-deficient diet-induced MASH in mice [29]. LDs were markedly increased in HepG2 cells overexpressing perilipin-2 [30]. Consistent with evidence that perilipin-2 promotes LD formation, resulting in lipid accumulation in the liver, we demonstrated that IF dramatically reduces hepatic perilipin-2 protein levels and pericentral perilipin-2 hepatocytes in HFD-fed mice. Taken together, these findings suggest that IF-induced inhibition or reduction in lipid uptake into hepatocytes could protect against HFD-induced hepatic damage.

Some studies have reported increased CD36/fatty acid translocase expression in the livers of mice and patients with MASLD [31,32]. Several clinical studies have demonstrated that patients with biopsy-proven MASLD had higher levels of hepatic CD36 expression than subjects with normal livers [20,33]. By contrast, hepatocyte-specific *cd36*-deleted mice exhibited enhanced insulin sensitivity and reduced MASLD [34]. Furthermore, because the *cd36* gene contains a PPAR element, its transcriptional expression can be modified by PPARs. Indeed, hepatic CD36 is increased by overexpressing PPAR-γ or inducing its activity with rosiglitazone [35,36]. Our study also demonstrated that HFD-induced CD36 and PPAR-γ expression levels were significantly reduced by IF. By contrast, the decrease in hepatic PPAR-α was reversed by IF. PPAR-α promotes β-oxidation of fatty acid in the liver, thereby reducing the synthesis of fatty acid and TG. PPAR-α-deficient mice fed an HFD exhibited a significant increase in body weight [37]. In light of this evidence, our results indicate that enhancing hepatic PPAR-α-mediated fatty acid oxidation may contribute to lipid accumulation and then promote progression to MASH.

The lipidomic analysis showed that hepatic PC and PE levels were reduced in MASH mice, but IF reversed these levels. PC and PE are two major phospholipids that are asymmetrically distributed in the plasma membrane [38]. In particular, PC is a PUFA compound that comprises 50% of Golgi membranes; 45% of plasma and inner mitochondrial membranes; and 60% of rough endoplasmic reticulum, nuclear, and outer mitochondrial membranes. Therefore, PC is a key regulator of cell membrane integrity and plays a role in the progression of MASH [12]. The findings of one study supported that decreased PC and PE levels adversely affect hepatocyte membrane integrity, resulting in liver damage, including lobular inflammation, hepatocyte ballooning, and fibrosis [39]. Therefore, we hypothesized that this augmented lipid accumulation in HFD-fed mice would promote hepatic inflammation and fibrosis. Consistent with the effects associated with MASLD progression in mice and humans [11], we found that IF reversed the reduced PC and PE levels in HFD-fed mice. Decreased levels of both PCs and PEs contribute to MASH by initiating inflammation or increasing LD fusion [12], Furthermore, Lee et al. demonstrated that soybean-derived PC treatment alleviated HFD-induced liver weight and hepatic lipid accumulation [40]. Taken together, our findings suggest that impaired hepatocyte membrane integrity caused by lipid alteration may play an important role in the development of MASLD and progression to MASH along with inflammation and fibrosis.

By contrast, we found that MASH mice exhibited lower levels of PUFA and higher levels of MUFA, suggesting impaired lipophagy. Omega fatty acids such as eicosapentaenoic acid or docosahexaenoic acid increase autophagic flux, as indicated by an elevated LC3BⅡ/Ⅰratio, and hence reduce lipotoxicity and increased accumulation in hepatocytes [9,41]. Activation of the autophagic flux leads to a decline in p62 expression, and by contrast, an increase in p62 reflects a decrease in the autophagic flux [42]. These findings support the theory that decreased autophagic flux impairs lipophagy and then results in a decreased efflux of lysosomes to the lumen of the CV [41]. We found that, in parallel with an increase in the LC3BⅡ/Ⅰratio in HFD-fed mice, the hepatic p62 protein level was also increased. TEM analysis supported the findings that increases in the number of LDs, lipolysosomes, and droplet fusion occurred in HFD-fed mice compared with those in ND mice. Many lipolysosomes fused to the plasma membrane of hepatocytes and effluxed into the CV in HIF-fed mice were comparable to those in HFD-fed mice. Therefore, these data suggest that the activation of the autophagic flux by IF may be essential for protection against hepatic lipid accumulation.

However, galectin-3 is produced by various cells, including immune cells, adipocytes, Kupffer cells, and hepatic stellate cells (HSCs) [43,44,45]. A recent study indicated that the galectin-3 inhibitor belapectin reduced hepatic fibrosis in patients with MASH [46]. We found that in addition to proinflammatory cytokines (TNF-α, IL-6, and MCP1) and HO-1 protein, HFD-fed mice had increased levels of galectin-3-positive Kupffer cells, whereas these inflammatory genes were reversed by IF. In our previous study, we demonstrated that LCN2 elevation promotes MMP9-mediated extracellular matrix degradation and MASH progression in HFD-fed *ob/ob* mice [26]. HSC activation-induced MMP9 production plays an important role in hepatic fibrosis through LCN2/STAT3-mediated signaling. In line with our finding that IF reduces hepatic LCN2, MMP9, and pSTAT3 expression in HFD-fed mice, LCN2 deletion and administration of a STAT3 phosphorylation inhibitor reduced inflammation and hepatic fibrosis [26]. In addition, lumican has been implicated in hepatic fibrosis. In a rodent model of carbon tetrachloride-induced hepatic fibrosis, lumican deficiency protected against hepatic fibrosis [47]. In this study, we also demonstrated that the increase in lumican protein in HFD-fed mice was significantly reversed by IF. Taken together, these data suggest a potential therapeutic strategy of IF for protection against hepatic inflammation and fibrosis.

Although we found significant effects, our study has several limitations. First, this mouse model showed the full spectrum of MASH pathological features, including steatosis, inflammation, and fibrosis. However, we did not assess the protective effects of short-term IF against MASH with diet-induced obesity. Second, this study did not directly assess the effects of lipid accumulation on lipophagy and lipolysis in primary hepatocytes. Third, follow-up was not performed to assess the sustained beneficial effects after the cessation of IF.

## Data Availability

The data presented in this study are available in this manuscript.

## Supplementary Materials

The following supporting information can be downloaded at: https://www.mdpi.com/article/10.3390/nu15214574/s1, Figure S1: IF attenuates body weight gain and improves insulin resistance in HFD-fed mice; Figure S2: IF changes the composition of FFAs in the liver of HFD-fed mice; Table S1: List of RT-PCR primers; Table S2: List of primary antibodies; Table S3: Total identified lipids using UPLC/Q-TOF MS.
