# Supplementary material for: Intermittent Fasting Attenuates Metabolic-Dysfunction-Associated Steatohepatitis by Enhancing the Hepatic Autophagy–Lysosome Pathway
Source: Nutrients. 2023 Oct 27;15(21):4574. doi: 10.3390/nu15214574 (PMC10649202; doi:10.3390/nu15214574)
Supplement: Supplementary file 1 [file nutrients-15-04574-s001.zip › nutrients-2688250-supplementary.pdf]

Supplementary figures

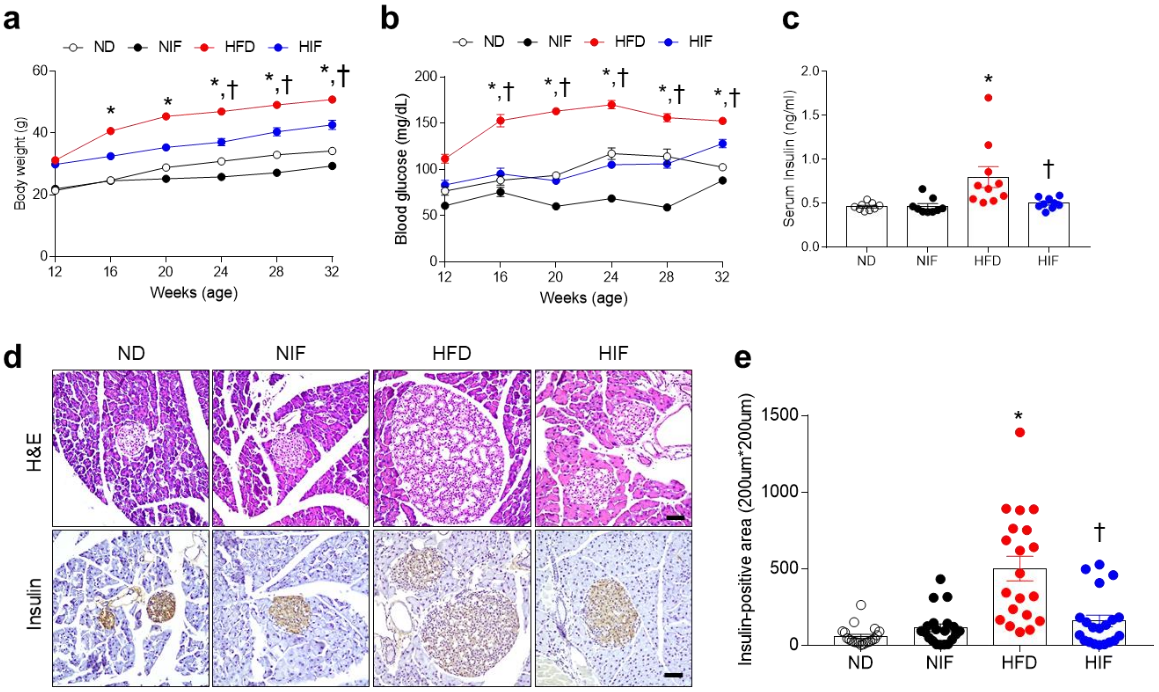

Supplementary Figure S1. IF attenuates body weight gain and improves insulin resistance in HFD-fed mice. (**a**) Body weight and (**b**) fasting blood glucose of mice after IF. (**c**) Serum insulin levels. (**d**) Representative microscopic images of mouse pancreatic sections stained with H&E and an anti-insulin antibody. (**e**) Percentage areas of insulin-positive cells. Data indicate the mean ± SEM. *$p < 0.05$ vs. ND-fed mice. Significance was determined with a two-way ANOVA. †$p < 0.05$ vs. HFD-fed mice. NIF: ND + IF, HIF: HFD + IF.

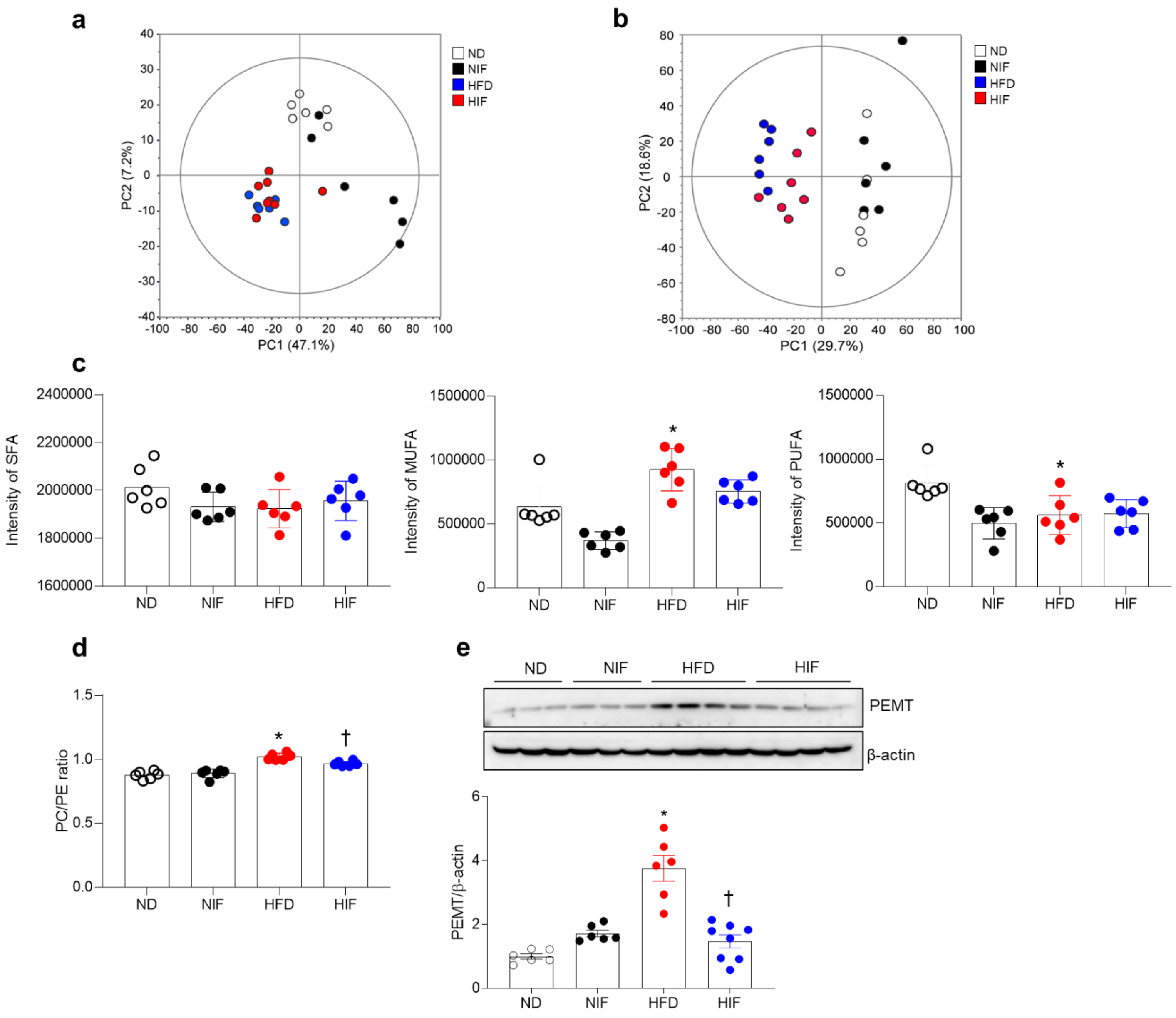

Supplementary Figure S2. IF changes the composition of FFAs in the liver of HFD-fed mice. (**a**,**b**) PCA of lipidomic data obtained using the positive-ion (**a**) and negative-ion modes (**b**). The indicated groups are presented in different colors. (**c**) Saturated fatty acid, MUFA, and PUFA. (**d**) PC/PE ratio. (**e**) Western blot analysis and quantification of PEMT protein in liver lysates. β-Actin was used as a loading control. Data are shown as the mean ± SEM. Significance was determined with the two-way ANOVA. $*p < 0.05$ *vs*. ND-fed mice. $\dagger p < 0.05$ *vs*. HFD-fed mice. NIF: ND + IF, HIF: HFD + IF.

Supplementary Table S1. List of RT-PCR primers.

Gene Name	Primer Sequences (Mouse)
TNF-α	Forward 5′ CCAGACCCTCACACTCAGATC 3'
	Reverse 5′ CACTTGGTGGTTTGCTACGAC 3'
IL-6	Forward 5′ AGTTGCCTTCTTGGGACTGA 3'
	Reverse 5′ TCCACGATTTCCCAGAGAAC 3'
MCP-1	Forward 5′ CCACTCACCTGCTGCTACTCA 3'
	Reverse 5′ TGGTGATCCTCTTGTAGCTCTCC 3'
IL-10	Forward 5′ CCAGGGAGATCCTTTGATGA 3'
	Reverse 5′ AACTGGCCACAGTTTTCAGG 3'
TGF-β1	Forward 5′ TGGAGCAACATGTGGAACTC 3'
	Reverse 5′ CAGCAGCCGGTTACCAAG 3'
Gapdh	Forward 5′ AAATGGTGAAGGTCGGTGTG 3'
	Reverse 5′ CATGTAGTTGAGGTCAATGAAGG 3'

Supplementary Table S2. List of primary antibodies.

Antibody	Company	Catalog No.	Dilution(s)	Applications	Source
Insulin	Abcam	ab7842	1:200	IHC	G. Pig
GS	Santa Cruz	sc-74430	1:200	IF	Mouse
Perilipin-2	Abcam	ab52356	1:2000, 1:200	WB, IF	Rabbit
CD36	Novus	NB400	1:1000	WB	Rabbit
FAS	Cell signaling	#3189	1:1,000	WB	Rabbit
SCD1	Cell signaling	#2438	1:1,000	WB	Rabbit
PPAR-α	Abcam	Ab8934	1:1000	WB	Rabbit
PPAR-λ	Santa Cruz	sc-7196	1:1000	WB	Rabbit
LC3B	Cell signaling	#83506	1:1000	WB	Mouse
p62	Sigma	P0067	1:1000	WB	Rabbit
LAMP1	Abcam	ab24170	1:1000	WB	Rabbit
LAL	Santa Cruz	sc-58374	1:1000	WB	Mouse
PEMT	LSBio	LS-C163519	1:1000	WB	Rabbit
Galectin-3	Santa Cruz	sc-23938	1:1000, 1:200	WB, IF	Rat
LCN2	R&D	AF1857	1:1000	WB	Goat
MMP9	Abcam	ab38898	1:1,000	WB	Rabbit
pSTAT3	Cell signaling	#9134	1:1,000	WB	Rabbit
STAT3	Cell signaling	#9139	1:1,000	WB	Mouse
Lumican	Abcam	ab168348	1:1000	WB	Rabbit
HO-1	Enzo	ADI-SPA-895	1:1,000	WB	Rabbit
F4/80	Santa Cruz	sc-377009	1:100	IF	Mouse
β-actin	Sigma	A5441	1:1,000	WB	Mouse

WB, Western blot; IF, immunofluorescence; IHC, immunohistochemistry.

Supplementary Table S3. Total identified lipids using UPLC/Q-TOF MS.

		Mean±SEM				Fold change	
Class	Name	ND	NIF	HFD	HIF	HFD/ND	HIF/HFD
FFA	FFA 12:0	3899.18±125.49	3518.57±91.19	3605.90±109.10	3887.84±178.56	0.92	1.08
	FFA 14:0	13864.82±924.13	9880.76±417.04	11695.16±506.01	13032.51±1158.34	0.84	1.11
	FFA 14:1	9791.47±1492.60	5016.24±965.84	6795.90±408.76	7724.39±795.12	0.69	1.14
	FFA 14:2	4086.95±591.68	2492.11±496.47	1384.27±101.68	2552.62±165.08	0.34	1.84
	FFA 16:0	669588.84±29816.90	500824.52±23625.33	592455.83±29582.12	594764.14±28123.95	0.88	1.00
	FFA 16:1	96422.62±13007.65	42261.26±3604.63	92655.14±7834.12	65758.44±2997.89	0.96	0.71
	FFA 16:2	8891.81±385.17	4656.34±863.60	3562.95±262.82	4652.14±304.44	0.40	1.31
	FFA 16:3	2122.19±92.95	873.98±188.03	922.05±66.40	1101.45±76.78	0.43	1.19
	FFA 18:0	1310726.43±28824.81	1400590.36±37093.90	1296821.70±7252.34	1324520.46±16310.14	0.99	1.02
	FFA 18:1	512871.26±60701.66	306936.61±24725.05	795813.18±59833.12	660141.64±37963.02	1.55	0.83
	FFA 18:2	359852.29±27207.51	211911.98±25557.23	188131.94±26037.37	218587.06±22480.85	0.52	1.16
	FFA 18:3	55195.55±3351.24	25489.49±4003.74	14785.06±1745.81	21038.53±2073.56	0.27	1.42
	FFA 18:4	3595.21±207.78	1240.27±371.26	658.44±101.50	1267.38±164.32	0.18	1.92
	FFA 20:0	4561.25±219.04	5476.90±391.82	7180.79±1159.37	6464.51±622.82	1.57	0.90
	FFA 20:2	13668.96±1360.81	11722.10±857.48	13957.59±1144.34	14794.73±686.10	1.02	1.06
	FFA 20:3	13396.48±1467.18	10121.46±816.92	19878.68±1929.33	15616.92±1216.99	1.48	0.79
	FFA 20:4	118110.33±9731.01	77299.24±8543.37	125525.52±11433.47	108928.22±7010.62	1.06	0.87
	FFA 20:5	55741.29±7223.09	15637.50±2651.87	14496.28±1753.61	16748.63±1818.83	0.26	1.16
	FFA 22:0	1903.63±317.23	2136.61±162.78	2674.11±534.18	2225.43±421.46	1.40	0.83
	FFA 22:1	4348.17±437.59	5154.06±543.41	7410.37±1078.03	4466.34±480.37	1.70	0.60
	FFA 22:2	948.40±108.65	1326.63±173.51	1392.67±158.68	936.34±106.68	1.47	0.67
	FFA 22:3	778.79±82.55	963.61±72.04	1473.30±145.83	1044.28±85.34	1.89	0.71
	FFA 22:4	17710.80±1397.23	15793.98±1117.09	29224.63±2005.36	22084.25±526.23	1.65	0.76
	FFA 22:5	27531.87±2162.72	17185.84±1894.20	21957.23±1951.84	21303.22±1413.07	0.80	0.97
	FFA 22:6	122045.84±7543.46	89017.95±10359.98	113644.51±14084.34	112610.91±8739.23	0.93	0.99
	FFA 24:0	188.57±39.09	195.95±10.15	120.05±14.78	333.59±222.17	0.64	2.78
	FFA 24:1	2820.66±440.04	2054.93±97.00	3503.90±319.13	1800.73±88.33	1.24	0.51
	FFA 24:2	419.67±43.48	351.21±44.23	211.65±36.15	169.35±23.89	0.50	0.80
	FFA 24:4	1860.20±367.78	2753.58±473.25	2479.16±196.78	2071.78±261.05	1.33	0.84
	FFA 24:5	4315.04±642.22	4419.16±797.98	2401.85±231.49	2590.40±174.50	0.56	1.08
	FFA 24:6	3435.63±558.00	4048.64±534.76	4876.35±428.24	4137.09±177.97	1.42	0.85
	FFA 26:0	5300.57±256.35	5788.11±170.84	5215.98±80.88	6247.31±1214.86	0.98	1.20
	FFA 28:0	1341.29±92.97	1509.50±24.78	1193.73±18.42	1899.27±618.55	0.89	1.59
	FFA 30:0	790.88±59.27	931.62±39.56	799.59±42.36	1212.87±383.87	1.01	1.52
	FFA 32:1	1239.06±153.80	1386.07±142.71	2353.33±198.30	2206.51±539.19	1.90	0.94
	FFA 32:0	379.13±18.75	423.77±52.43	917.03±39.74	866.59±129.43	2.42	0.94
	FFA 34:0	448.38±18.93	562.86±16.71	629.99±17.91	630.27±57.84	1.41	1.00
	FFA 34:1	2997.98±339.39	3119.61±379.03	6944.07±487.96	5223.44±719.05	2.32	0.75
	FFA 36:1	2696.31±218.65	2694.37±252.82	7199.54±365.49	5056.04±676.80	2.67	0.70
	FFA 38:1	314.90±50.68	1078.10±217.13	1887.75±127.89	1608.28±250.71	5.99	0.85
Cer	Cer d42:2	15442.56±459.18	16027.75±338.89	15844.64±256.95	15819.60±456.30	1.03	1.00
GlcCer	GlcCer d36:0	46040.13±1011.14	46094.56±522.53	45503.56±438.42	45525.15±153.03	0.99	1.00
CL	CL 72:3	89576.14±4252.73	81681.93±1838.20	53678.91±973.56	63913.12±926.59	0.60	1.19
	CL 72:4	1691.66±84.03	1713.73±58.63	1256.65±50.44	1394.91±37.50	0.74	1.11
	CL 72:7	783.03±47.00	642.95±53.78	497.56±28.87	547.88±19.16	0.64	1.10
SM	SM d34:1	2768.15±223.49	3017.95±82.64	2075.27±189.34	3170.44±175.39	0.75	1.53
	SM d34:2	3546.17±127.39	3484.03±119.85	3618.91±116.62	3273.80±39.14	1.02	0.90
	SM d36:1	65670.68±2312.89	66995.57±2703.68	96564.08±6000.37	137690.86±10415.73	1.47	1.43
	SM d40:1	4502.48±661.34	6178.49±743.39	2500.44±418.91	4808.71±648.35	0.56	1.92
	SM d42:1	1388199.05±55509.92	1510735.05±55711.85	783237.89±20384.03	985283.51±29472.98	0.56	1.26
	SM d42:2	4396.92±508.03	4780.25±163.83	2161.36±244.46	3188.51±341.08	0.49	1.48

**LysoPA**	LysoPA 16:0	8787.78±457.66	6483.75±736.94	8090.29±930.01	8077.61±550.57	0.92	1.00
	LysoPA 18:0	2956.38±120.26	3198.61±127.27	3490.45±14.88	3630.44±81.25	1.18	1.04
	LysoPA 18:1	1309.54±85.35	1326.12±34.46	1330.57±34.81	1262.29±17.81	1.02	0.95
**LysoPC**	LysoPC 16:0	138029.16±14469.30	139925.77±4723.72	139234.80±3999.35	130748.83±1621.34	1.01	0.94
	LysoPC 16:1	5480.60±1047.92	4883.47±410.79	3774.67±122.03	3669.46±106.84	0.69	0.97
	LysoPC 18:0	111587.87±7485.16	123062.42±4918.81	127157.17±3514.99	142795.80±1061.83	1.14	1.12
	LysoPC 18:1	6783.95±441.58	8145.20±646.73	11637.01±323.99	9545.27±220.71	1.72	0.82
	LysoPC 18:2	28613.36±2356.21	30991.94±1638.94	15584.22±744.82	21776.07±566.04	0.54	1.40
	LysoPC 20:0	1647.67±217.98	2674.08±462.64	1465.56±202.42	1541.48±229.08	0.89	1.05
	LysoPC 20:1	1052.58±160.97	1163.97±167.24	1108.46±76.66	865.61±36.74	1.05	0.78
	LysoPC 20:3	1369.56±156.01	2235.21±247.50	2335.57±91.49	2263.20±157.85	1.71	0.97
	LysoPC 20:4	28855.71±2699.89	28982.03±1278.87	30831.43±1124.37	33715.84±1094.93	1.07	1.09
	LysoPC 22:0	373.78±34.06	610.29±85.96	156.53±18.06	314.29±22.88	0.42	2.01
	LysoPC 22:6	10334.72±1043.00	11241.45±673.03	11082.78±430.89	11978.04±311.24	1.07	1.08
	LysoPC 24:0	565.11±60.21	647.76±31.33	166.33±14.34	480.98±34.48	0.29	2.89
	LysoPC O-16:0	104.80±32.49	189.37±37.13	160.34±29.81	233.38±25.76	1.53	1.46
**LysoPE**	LysoPE 18:0	15462.80±2558.33	22614.25±1481.10	20179.39±1021.07	26149.06±2584.39	1.31	1.30
	LysoPE 18:1	8568.71±2143.08	8772.87±860.89	8509.46±320.11	8998.71±240.52	0.99	1.06
	LysoPE 18:2	3025.23±629.32	1965.45±167.89	781.59±116.48	1342.18±70.33	0.26	1.72
	LysoPE 20:4	7288.07±919.67	7243.72±358.91	6629.46±458.96	7685.77±227.05	0.91	1.16
**PC**	PC 30:0	37894.81±1185.50	40193.22±1373.34	21170.87±1593.40	29446.24±660.40	0.56	1.39
	PC 32:0	3933.71±334.07	4401.88±215.33	2142.66±171.36	3237.09±296.32	0.54	1.51
	PC 32:1	482183.18±5170.81	478597.98±4231.44	419020.10±4140.17	450654.53±5502.30	0.87	1.08
	PC 32:2	39933.73±1644.67	33663.63±3254.73	17123.04±765.48	18400.81±792.34	0.43	1.07
	PC 34:0	229139.31±5683.42	267405.51±8273.37	215077.42±5427.37	247980.54±5893.22	0.94	1.15
	PC 34:1	20567.99±1865.69	22772.74±809.45	17674.37±1686.66	25166.31±1895.14	0.86	1.42
	PC 34:2	84205.91±7759.01	88241.60±3567.27	25802.42±3632.34	57953.57±4859.56	0.31	2.25
	PC 34:3	163439.21±10043.95	138196.30±7621.75	103651.88±5384.07	103768.10±4522.08	0.63	1.00
	PC 34:4	9654.32±685.46	4457.90±513.64	135.50±58.71	353.70±87.92	0.01	2.61
	PC 36:1	2827.19±184.64	2884.99±158.13	2326.21±193.30	3317.41±190.81	0.82	1.43
	PC 36:2	1486480.48±38874.79	1569245.96±29511.16	1092743.88±31819.72	1432524.91±24163.70	0.74	1.31
	PC 36:3	12597.22±1210.23	12105.91±958.99	3508.98±414.87	7168.60±656.44	0.28	2.04
	PC 36:4	49651.66±4471.35	51550.47±1292.95	30280.86±2523.60	54068.00±4852.19	0.61	1.79
	PC 36:5	99246.76±8356.13	86512.48±5886.41	9799.64±1261.13	23237.98±1539.35	0.10	2.37
	PC 38:3	1873.78±228.43	1974.07±97.49	1426.53±132.57	2194.75±282.03	0.76	1.54
	PC 38:4	3027421.24±44426.65	3136538.59±39066.66	3168679.18±63911.02	3296689.04±25634.19	1.05	1.04
	PC 38:5	13532.90±1372.84	13511.03±536.62	6290.38±590.34	11247.88±1099.64	0.46	1.79
	PC 38:6	2057251.56±47282.70	2175314.21±43367.24	1893740.48±16714.58	2134083.01±30103.73	0.92	1.13
	PC 38:7	27915.86±3289.78	32148.38±1335.80	3382.29±563.38	11375.73±893.21	0.12	3.36
	PC 40:3	34983.78±1005.09	40386.81±2319.71	29686.98±602.50	30867.90±1468.91	0.85	1.04
	PC 40:4	33278.79±444.86	34491.10±1175.13	37820.13±2332.56	39903.08±663.70	1.14	1.06
	PC 40:5	44348.63±1347.78	42200.62±1779.94	40768.23±1218.29	30890.78±810.08	0.92	0.76
	PC 40:6	706406.65±37839.68	702821.75±27714.93	632132.94±16121.36	744041.05±12723.54	0.89	1.18
	PC 40:7	473997.86±16088.98	459028.31±16193.63	418720.63±6789.86	442978.83±6966.24	0.88	1.06
	PC 40:8	110821.85±12758.47	136664.58±10323.35	65796.58±1477.05	106429.40±4793.60	0.59	1.62
	PC 42:5	12120.85±573.11	13146.86±578.23	9980.76±239.44	9500.36±240.20	0.82	0.95
	PC 42:6	27548.95±1541.49	37204.67±3264.97	23400.08±1411.73	24588.66±2308.37	0.85	1.05
	PC 42:8	5583.41±329.39	5950.72±193.83	5352.04±113.89	5613.03±89.42	0.96	1.05
	PC 42:10	26774.48±4719.40	54872.63±5607.98	26652.99±1072.90	48286.94±2543.89	1.00	1.81
**PC-O**	PC O-34:1	97582.06±3325.17	97744.72±2994.46	91084.60±2893.75	84328.41±1413.95	0.93	0.93
	PC O-34:2	541.23±31.33	543.06±30.49	607.07±42.15	536.91±27.61	1.12	0.88
	PC O-36:2	190823.37±9011.51	184804.53±10617.29	112137.36±4073.03	162259.22±5010.01	0.59	1.45
	PC O-36:3	8918.12±224.48	9591.39±259.00	10160.53±232.05	9139.23±171.14	1.14	0.90
	PC O-36:4	13349.47±243.12	13849.03±439.79	15849.75±285.38	14870.98±337.77	1.19	0.94
	PC O-40:7	7316.37±280.50	7195.36±185.94	7960.27±287.57	6860.18±76.06	1.09	0.86

**PE**	PE 32:2	1588.77±156.56	426.81±68.04	9.99±3.93	36.39±8.82	0.01	3.64
	PE 34:1	128081.57±17796.76	100718.91±3402.53	85876.79±3406.29	87019.52±1859.42	0.67	1.01
	PE 34:3	97244.00±7359.19	52052.88±3305.09	11012.95±467.02	18451.16±589.10	0.11	1.68
	PE 36:0	15703.41±598.23	19219.68±854.24	13200.31±671.89	17292.04±883.94	0.84	1.31
	PE 36:1	141826.30±17158.88	136607.58±3541.80	105072.29±6794.54	134339.93±5756.00	0.74	1.28
	PE 36:2	1166238.86±62683.24	1161537.69±41674.46	467707.19±28561.44	862000.76±22018.47	0.40	1.84
	PE 36:3	1109763.48±50325.77	913833.91±31175.97	401105.39±20962.54	616671.61±13630.76	0.36	1.54
	PE 36:5	67428.50±5139.32	62147.86±5079.99	28374.61±1426.33	43233.36±2560.20	0.42	1.52
	PE 38:1	72332.99±2343.48	69698.81±2457.02	90131.04±3051.59	91143.49±1551.43	1.25	1.01
	PE 38:3	262640.89±18760.57	318688.76±10982.66	226671.78±8124.03	276176.50±10963.25	0.86	1.22
	PE 38:4	3288071.62±55941.78	3726283.71±34755.93	3075902.07±27974.73	3513669.57±22239.95	0.94	1.14
	PE 38:6	2475956.59±29930.28	2540122.50±27742.72	2108650.04±40094.04	2392446.79±32743.62	0.85	1.13
	PE 38:7	121443.58±4738.17	83640.60±4651.23	38447.91±1717.67	44373.30±2779.20	0.32	1.15
	PE 40:3	69137.99±3142.76	77912.68±2989.83	96634.59±1777.72	90930.09±5518.60	1.40	0.94
	PE 40:4	174619.37±4325.32	205775.94±5416.37	158993.52±8097.94	183904.52±5360.77	0.91	1.16
	PE 40:5	66415.30±1674.34	65212.95±2632.83	74318.36±2719.72	72069.88±2128.92	1.12	0.97
	PE 40:6	63144.78±1098.64	65422.03±2287.13	41076.36±1437.50	44722.36±1217.28	0.65	1.09
	PE 40:7	1204422.62±19698.20	1144324.86±36425.89	1077235.44±34658.32	1143673.38±23862.46	0.89	1.06
	PE 40:8	80190.11±7162.64	98450.26±4052.70	35959.41±1633.60	60354.25±2197.27	0.45	1.68
	PE 42:8	14301.81±590.78	16031.89±517.75	15560.72±527.45	13189.37±340.16	1.09	0.85
	PE 42:9	8392.22±436.01	12253.39±706.99	7297.91±202.08	9466.75±299.23	0.87	1.30
	PE 42:10	5378.37±707.99	11957.94±1625.69	3455.57±227.57	8049.45±551.28	0.64	2.33
**PE-O**	PE O-36:5	314014.32±7195.41	352645.86±9897.82	303389.37±10252.97	296673.38±7201.34	0.97	0.98
	PE O-38:2	18237.41±760.30	19575.92±306.34	18944.79±589.12	18431.14±582.54	1.04	0.97
	PE O-38:4	21231.63±1178.13	19647.88±542.79	12382.36±338.15	15041.44±608.48	0.58	1.21
	PE O-38:5	252182.75±6542.96	288899.07±6905.24	325466.16±8561.95	409733.99±12477.23	1.29	1.26
	PE O-40:4	67365.08±1462.98	79537.15±1827.78	90142.70±1766.53	86527.53±2174.33	1.34	0.96
	PE O-40:6	57040.89±1099.78	59178.29±811.72	53496.91±600.14	62749.97±1564.44	0.94	1.17
**PG**	PG 36:2	11063.02±650.90	13008.16±913.02	5374.60±432.57	13257.04±1017.08	0.49	2.47
	PG 36:3	18474.37±944.74	20572.44±1933.10	30333.39±991.69	30451.90±2376.45	1.64	1.00
	PG 36:4	14151.78±1404.01	11837.62±2020.18	6530.40±343.33	10233.75±900.61	0.46	1.57
	PG 38:4	4850.65±257.52	5302.89±375.55	4993.70±185.17	6822.42±487.70	1.03	1.37
	PG 38:5	7559.20±606.60	7811.06±531.07	22007.56±1241.20	16668.60±1011.44	2.91	0.76
	PG 38:6	5881.22±367.71	4874.33±439.90	6129.47±341.68	6287.62±390.83	1.04	1.03
	PG 40:6	1776.06±146.90	1854.37±276.42	1717.14±171.11	1730.13±185.91	0.97	1.01
	PG 40:7	25403.07±2905.55	33150.07±2373.66	75256.61±5436.44	68995.59±4609.63	2.96	0.92
	PG 40:8	38357.49±3320.44	39429.19±3842.92	38233.60±1769.50	47639.24±2171.48	1.00	1.25
	PG 42:8	1087.58±178.87	641.63±135.92	2361.68±395.22	2023.77±180.88	2.17	0.86
	PG 42:10	9131.33±1365.62	8930.69±820.11	18517.82±1570.55	17266.04±1096.62	2.03	0.93
	PG 44:11	1697.99±625.31	3719.54±935.83	7040.09±964.18	6503.97±459.04	4.15	0.92
	PG 44:12	41180.39±6848.81	53186.37±6256.86	80012.16±6346.52	79777.00±4122.20	1.94	1.00
**PI**	PI 38:4	84340.25±6873.82	128224.00±10807.15	132763.40±7233.61	125740.63±17272.75	1.57	0.95
	PI 38:5	5073.48±770.11	6872.28±1148.49	5812.51±461.24	4416.72±761.57	1.15	0.76
**PS**	PS 36:4	2167.51±343.70	2776.70±345.75	1861.95±182.74	2097.80±413.93	0.86	1.13
	PS 38:6	2278.55±227.52	2635.29±275.46	1571.66±139.31	1683.86±196.19	0.69	1.07
	PS 40:6	12371.38±377.68	16168.27±1297.26	11448.76±828.97	13253.38±1254.37	0.93	1.16
	PS 44:12	4361.72±176.88	4829.58±148.60	1997.81±171.95	3304.70±148.90	0.46	1.65
**DAG**	DAG 42:6	23504.27±333.25	24929.00±734.55	22782.00±215.72	22944.80±363.85	0.97	1.01

	TAG 42:0	96842.48±1842.96	100088.38±1253.20	94422.05±1107.45	94060.40±703.16	0.98	1.00
	TAG 46:0	124704.18±4013.91	126235.78±1658.70	114653.33±1200.95	114931.87±884.55	0.92	1.00
	TAG 46:1	86588.88±3805.91	85355.57±1225.76	79449.02±1001.00	80587.19±916.86	0.92	1.01
	TAG 46:2	20319.87±1247.63	19327.05±310.17	17950.08±184.08	17992.99±390.93	0.88	1.00
	TAG 46:4	7770.14±218.79	7662.76±191.83	7064.22±97.58	7294.16±83.34	0.91	1.03
	TAG 48:0	75991.15±2755.51	75701.32±1389.35	75969.49±928.28	75374.16±1177.54	1.00	0.99
	TAG 48:1	97545.18±6731.45	83902.81±2609.40	110252.43±2474.97	101335.38±6423.93	1.13	0.92
	TAG 48:2	84536.33±6262.84	65573.26±2193.84	79280.15±2235.89	74007.67±3820.08	0.94	0.93
	TAG 48:3	18543.51±2163.99	12194.79±816.97	13039.22±385.23	12605.99±598.95	0.70	0.97
	TAG 48:4	5855.19±603.37	3793.78±191.02	3767.01±92.00	3790.96±183.54	0.64	1.01
	TAG 50:0	51431.86±1218.67	51309.67±732.59	51965.65±950.59	51635.14±470.32	1.01	0.99
	TAG 50:1	105290.30±9811.47	75640.01±11123.74	376112.64±10900.91	291694.29±41716.49	3.57	0.78
	TAG 50:2	363865.58±37905.30	193237.72±38442.67	651593.52±31879.67	520694.86±72066.63	1.79	0.80
	TAG 50:3	197984.95±28235.53	69887.78±20251.18	177853.81±11529.84	150747.70±20703.88	0.90	0.85
	TAG 50:4	54867.67±8574.20	21013.01±4592.35	31087.66±1874.19	29251.90±2722.58	0.57	0.94
	TAG 50:8	91387.31±2989.65	85890.80±1302.13	83340.81±877.21	83480.77±1153.90	0.91	1.00
	TAG 52:0	29490.63±566.23	28862.36±494.88	28384.98±353.67	28562.70±319.37	0.96	1.01
	TAG 52:1	28701.76±2657.23	26518.40±2158.20	86390.68±4200.68	77701.42±7908.61	3.01	0.90
	TAG 52:2	446909.84±61251.53	227225.79±65558.14	1968004.85±88571.25	1626254.20±186917.06	4.40	0.83
	TAG 52:3	1240209.48±103448.07	603611.53±220916.13	1989310.86±132010.78	1936975.65±180241.30	1.60	0.97
	TAG 52:4	1243805.27±139818.40	541096.95±207827.35	772512.73±86753.21	897559.75±85246.27	0.62	1.16
**TAG**	TAG 52:5	189855.29±28071.58	68235.08±26144.22	74897.45±7799.05	89584.99±7847.54	0.39	1.20
	TAG 52:6	16336.06±1519.67	9240.24±1535.86	10350.57±454.21	11162.43±534.33	0.63	1.08
	TAG 54:0	11260.48±386.22	11209.97±272.31	10287.73±196.21	10562.22±310.76	0.91	1.03
	TAG 54:1	19196.69±3037.85	18498.49±1265.42	27120.43±2001.15	26818.56±2112.61	1.41	0.99
	TAG 54:2	43641.43±5283.37	35752.72±4463.61	170409.78±10220.26	142329.50±16776.42	3.90	0.84
	TAG 54:3	174305.44±16682.35	106174.23±24050.84	678385.10±54823.92	554586.85±69654.89	3.89	0.82
	TAG 54:4	435645.60±42167.18	232817.00±66822.96	670638.57±65453.20	672532.97±66543.03	1.54	1.00
	TAG 54:5	59912.36±3265.32	39541.81±6083.00	124660.24±3677.03	108788.73±13088.46	2.08	0.87
	TAG 54:6	201509.70±29960.89	89738.21±31155.77	53499.78±5543.88	72466.03±6713.30	0.27	1.35
	TAG 54:7	36630.65±6042.60	15032.32±5116.49	6494.37±515.41	8464.74±655.04	0.18	1.30
	TAG 54:8	12162.11±1580.84	5356.13±1269.13	4820.94±271.71	5203.65±325.47	0.40	1.08
	TAG 56:0	4818.80±270.96	4836.00±125.79	4198.58±164.29	4613.34±129.40	0.87	1.10
	TAG 56:4	28551.99±2252.53	20115.35±4768.56	75592.17±5996.79	65527.68±7505.26	2.65	0.87
	TAG 56:6	8719.59±895.01	5240.27±1430.95	13798.78±1094.32	12512.78±967.47	1.58	0.91
	TAG 56:7	106285.89±12013.93	60391.01±19363.68	189867.15±14965.69	164923.99±16452.37	1.79	0.87
	TAG 56:8	182711.89±20667.12	83966.70±29112.96	93429.84±11359.53	105861.90±9389.49	0.51	1.13
	TAG 56:9	16363.98±2328.18	5536.79±2121.79	5152.81±503.62	5515.15±589.32	0.31	1.07
	TAG 58:7	4181.61±179.47	3573.82±531.79	8199.49±782.23	6655.26±368.64	1.96	0.81
	TAG 58:8	36525.11±4132.16	24707.57±6526.10	50935.72±3843.09	45898.58±4346.02	1.39	0.90
	TAG 58:9	56636.93±8082.00	31248.40±10099.21	29959.34±3441.03	34566.14±3244.16	0.53	1.15
	TAG 58:10	20648.32±3374.33	9303.71±3475.29	4262.93±688.14	6285.07±702.08	0.21	1.47
	TAG 58:11	3373.27±457.27	1312.36±462.76	487.83±41.53	874.80±134.75	0.14	1.79
